# The BIOMONITOR III Injectable Cardiac Monitor: Clinical Experience with a Novel Injectable Cardiac Monitor

**DOI:** 10.3390/jcm11061634

**Published:** 2022-03-16

**Authors:** Nico Reinsch, Anna Füting, Dennis Höwel, Kars Neven

**Affiliations:** 1 Department of Electrophysiology, Alfried Krupp Krankenhaus, 45131 Essen, Germany; anna.fueting@krupp-krankenhaus.de (A.F.); dennis.hoewel@krupp-krankenhaus.de (D.H.); kars.neven@krupp-krankenhaus.de (K.N.); 2 Department of Medicine, Witten/Herdecke University, 58448 Witten, Germany

**Keywords:** BIOMONITOR III, implantable cardiac monitor, implantable loop recorder, signal quality, R-wave sensing

## Abstract

Background: Injectable cardiac monitors (ICMs) are leadless subcutaneous devices for long-term monitoring of arrhythmias. The BIOTRONIK BIOMONITOR III is a novel ICM with a miniaturized profile, long sensing vector, and simplified implantation technique. Methods: R-wave amplitude was recorded immediately after implantation, the day after implantation, and after 3 months. Follow-up was scheduled after 3 months or after an event. All data from the ICM were retrieved. The anatomical position of the ICM was determined post-implantation and after 3 months. A patient questionnaire was conducted after 3 months. Results: In 36 patients (mean age 67 ± 13 years; 40% male) an ICM was inserted. Six patients were not included in the final analysis. The median time from skin cut to wound closure was 6 [IQR 5–7] minutes. Mean R-wave amplitude increased over time (0.73 ± 32 mV vs. 0.78 ± 0.38 mV vs. 0.81 ± 0.39 mV; *p* = ns). Three months after implantation, the ICM was in an anatomically stable position. In 14 (47%) patients, true episodes were detected. False arrhythmia alerts were detected in 13 (43%) patients. The total number of false detections was low, and the patient satisfaction rate was high. Conclusion: Implantation of the novel BIOMONITOR III is fast and uncomplicated; its sensing characteristics are excellent and improve over time, and patient satisfaction is high.

## 1. Introduction

Injectable cardiac monitors (ICMs) are subcutaneous, single-lead, electrocardiographic (ECG) monitoring devices capable of storing and transmitting ECG data automatically in response to a significant brady- or tachyarrhythmia. They are used as a diagnostic tool in patients with recurrent, unexplained episodes of palpitations or syncope, for long-term monitoring in patients at risk of atrial fibrillation (AF), and to guide clinical management in patients with known AF. In addition, ICMs can be used in follow-up monitoring after catheter ablation [1,2,3]. Therefore, the European Society of Cardiology guidelines have strengthened the implantation of ICMs [4]. However, the detection of arrhythmias by ICMs is limited by false positives and artifacts, requiring technological advancement to improve the diagnostic yield [5,6,7]. A miniaturization of the ICM is desired in order to increase the acceptance of the ICM by patients and physicians, but this conflicts with the fact that a larger electrode spacing can improve the signal quality and, thus, the diagnostic reliability by reducing undersensing and noise artifacts [3,8,9,10]. The BIOMONITOR III (BIOTRONIK SE & Co., KG, Berlin, Germany) is a novel ICM combining a long sensing vector with a miniaturized profile; it promises a simple implantation procedure with a specially designed fast insertion tool (FIT) for pocket formation and ICM placement in a single step [11]. The aim of this study was to investigate the reliability of sensing quality, the detection performance, and the anatomical stability after insertion of the ICM, as well as the associated patient satisfaction.

## 2. Materials and Methods

### 2.1. Study Subjects

We performed a single-center, prospective, observational study to evaluate the post-procedural sensing quality of the ICM in consecutive patients who underwent ICM implantation, the anatomical stability after insertion, and the patients’ contentment concerning discomfort, feeling of safety, and technical difficulties. Data collection regarding baseline characteristics, procedural data, complications and, furthermore, the detected cardiac rhythm events and signal amplitudes, was performed during in-house and outpatient follow-up visits. We included patients who performed their follow-up visits on a regular basis, were at least 18 years old, and had at least one of the following indications for long-term cardiac monitoring: (1) symptoms of palpitations, pre-syncope, or syncope suggestive of an underlying cardiac arrhythmia, (2) cryptogenic stroke, or (3) high risk of developing a clinically relevant cardiac arrhythmia. Patients were excluded when they already had an implantable cardiac device, had participated in another interventional clinical investigation, were pregnant, or had a life expectancy of less than 12 months. Informed consent was obtained from all subjects involved in the study. The study was conducted in accordance with the ethical principles of the Declaration of Helsinki. The study protocol was approved by the ethics committee of Witten/Herdecke University (Witten, Germany) with approval number 130/2019.

### 2.2. Device Specifications

The BIOMONITOR III ICM is 77.5 mm long (device with antenna), 8.3 mm wide, and 4.3 mm thick, with a weight of 5 g and a volume of 1.97 cm^3^; its cross-sectional profile is comparable to that of other ICMs [9]. The sensing vector of ≈70 mm is ~50% longer than in comparable ICMs, promising larger R-wave amplitudes [11]. The ICM’s housing is made of sealed biocompatible titanium coated in silicone, while the flexible antenna is made of silicone and has a fractally coated electrode on its tip. At the end of the housing there is an opening in the coating, so that the metal housing forms the antipole to the lead tip. The lead’s conductor is also the antenna for Home Monitoring^®^ (BIOTRONIK SE & Co., KG, Berlin, Germany) remote monitoring function; it can store episodes with a maximum overall length of 60 min. The maximum storage period for an individual episode is 60 s, while a total of 56 episodes with a length of at least 40 s can be stored automatically. There is also the possibility of storing a total of four recordings, which are triggered by the patient, with a duration of 7.5 min. Possible detection types include atrial tachycardia, high ventricular rate, asystole, bradycardia, and a sudden rate drop. The ICM analyzes the heart rhythm according to the rate and regularity of R-waves. Noise is defined by cycle lengths below 180 ms, which limit the rhythm interpretation. Noise burden is characterized as the percentage of a 24 h period during which very fast signals inhibit rhythm analysis. The ICM offers simple programming through predefined, indication-related programming and improved signal quality through optimized filtering; it has an expected battery life of 4 years, and is compatible with magnetic resonance imaging for full-body scans up to 3 tesla.

### 2.3. BIOMONITOR III Insertion

**Figure 1:**
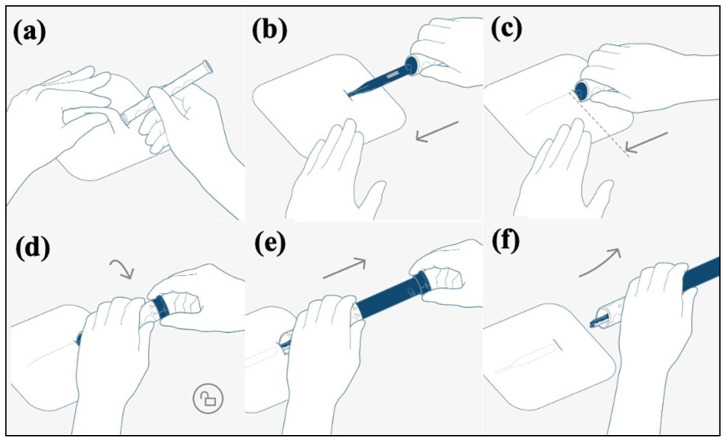
Insertion procedure: (**a**) A skin fold is formed and the cutting tool produces a defined incision. (**b**,**c**) The fast insertion tool forms the device pocket. (**d**) The tool is unlocked. (**e**,**f**) The tool holds the device in place while it is withdrawn from the pocket. Reprinted with permission from BIOTRONIK SE & Co., KG, Berlin, Germany.

The ICM insertion was carried out following the manufacturer’s instructions by an experienced cardiologist. The procedure took place in the cardiac catheterization lab while using sterile techniques. Pre-implantation, local anesthetic (20 mL of scandicain 1%) was administered. The implantation procedure is shown in Figure 1. The insertion place for the ICM was in the left parasternal region between the suprasternal notch and the left nipple. In order to obtain a high signal amplitude, it is important that the sensing vector is as close to parallel to the heart’s electrical axis as possible. Therefore, the ICM is preferentially implanted at a 45° angle to the sternum. To assure minimal device movement due to positional changes or body or arm movement, the ICM can alternatively be implanted in a parallel or inframammary fashion, depending on the operator’s discretion. Using the custom scalpel, a surgical cut for the device pocket was made. Subsequently, the FIT OneStep insertion tool (BIOTRONIK SE & Co., KG, Berlin, Germany) that forms the pocket and holds the ICM was inserted and advanced into the subcutaneous tissue layer parallel to the surface of the chest. Reaching the correct position, the ICM was released, and the insertion tool was removed from the body. Afterwards, the incision was closed in one layer using absorbable suture material completed with Steri-Strips (3M, St. Paul, MN, USA), depending on the operators’ discretion.

### 2.4. Data Evaluation and Consecutive Follow-Up

**Figure 2:**
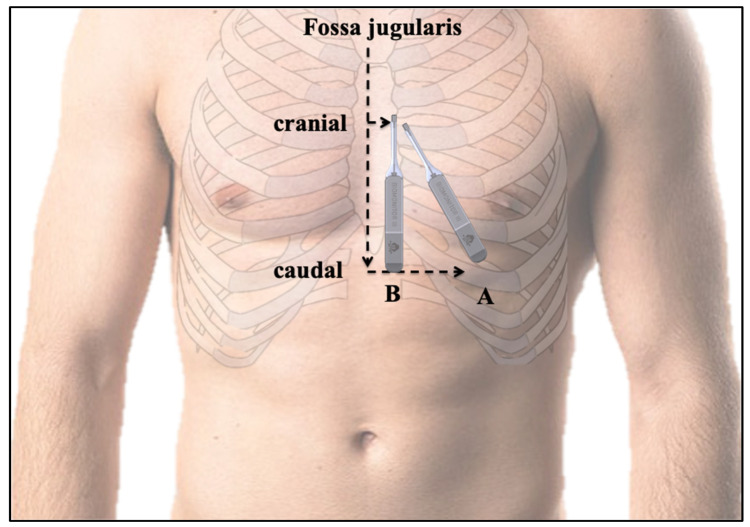
Anatomical position of the BIOMONITOR III: (A) Recommended and (B) alternative anatomical positions of the BIOMONITOR III in the present study.

The total skin-to-skin implantation time was recorded. Immediately after implantation, the R-wave data were collected to ensure the correct placement of the ICM. The day after insertion, measurement of sensing was repeated, the anatomical position was determined according to a prespecified scheme (Figure 2), and the patients received detailed instructions from a device specialist nurse. After three months, follow-up was scheduled and data from the ICM were downloaded, with documentation of all episodes, the R-wave amplitude, and noise burden. Furthermore, the anatomical position of the ICM was reassessed. If possible relevant clinical or arrhythmogenic events occurred, earlier appointments were arranged individually. 

### 2.5. Patient Survey

Using the patient survey, information concerning patients’ discomfort, pain, paresthesia, limitations in daily activities, handling of the Home Monitoring^®^ remote monitoring system, and overall satisfaction was collected. Eleven questions were answered, using a score from 0 to 10. The questionnaire was answered during the 3 months follow-up. 

### 2.6. Statistical Analysis

Data are shown as the mean ± standard deviation. A one-way ANOVA test was used to compare R-wave amplitudes and ICM positions between implantation and follow-up. A *p*-value < 0.05 was considered statistically significant.

## 3. Results

### 3.1. Patient Characteristics 

Between November 2019 and February 2021, we successfully inserted the BIOMONITOR III ICM into 36 patients. Four patients did not attend their follow-up appointment. One patient died without association with the implantation. In one female 90-year-old patient, the ICM protruded 2 weeks post-implantation—probably because of distinct cachexia. Thirty patients were included in our final analysis at three months after implantation. Indications for ICM implantation were syncope in 24 (80%), AF monitoring in 1 (4%), and cryptogenic stroke in 5 patients (16%). The mean follow-up time was 107 ± 59 days. The study participation of the last patient ended in June 2021. Baseline characteristics are shown in Table 1. Due to the small sample size, no sex-based differences could be detected.

### 3.2. Insertion Procedure Results and Position of the ICM

The implantation success rate was 100%. All 36 insertions were performed in the cardiac catheterization lab, and succeeded on the first attempt. The median time from skin cut to last suture was 6 ± 1 min (IQR: 5–7 min). No systematic or local antibiotics were applied. There were no pocket infections during the study. Local anesthesia was used in all patients. There were no periprocedural complications. All ICMs except one were implanted in the left parasternal region at an angle of approximately 45° or a parasternal orientation. One ICM was implanted in a mid-axillary position due to patient preference and a low BMI (17.7 kg/m^2^). The day after implantation and at the 3-month follow-up, the ICM position was assessed [12], demonstrating an overall anatomically stable position of the ICM. The vertical distance from the fossa jugularis to the lowest part of the device was 14.5 ± 2.5 cm directly after implantation and 14.5 ± 2.7 cm (*p* = 0.93) at the follow-up appointment. The horizontal cranial sternal to ICM distance was 6 ± 2.9 cm, and 5.7 ± 3.1 cm after three months (*p* = 0.11). The horizontal caudal sternal to ICM distance was 6.0 ± 9.1 cm and 5.7 ± 9.8 cm, respectively (*p* = 0.7).

### 3.3. Signal Quality

R-wave analysis was performed in 30 patients. The sensing parameters are shown in Table 2. Directly after the implantation, sensing was 0.73 ± 0.32 mV, ranging from 0.27 mV (minimum) to 1.47 mV (maximum). The following day sensing values were 0.78 ± 0.38 mV (*p* = 0.236), ranging from 0.25 mV (minimum) to 1.52 mV (maximum). After a mean follow-up of 107 ± 59 days, mean sensing was 0.81 ± 0.39 mV (*p* = 0.336), ranging from 0.20 (minimum) to 2.00 mV (maximum). Minimal R-wave sensing was 0.2 mV and 0.3 mV, respectively. The median noise burden in all patients was <2%. In no patient was the diagnostic power reduced because of a high noise burden. 

### 3.4. Arrhythmia Episodes Registered via Home Monitoring^®^ and In-House Follow Up

**Figure 3:**
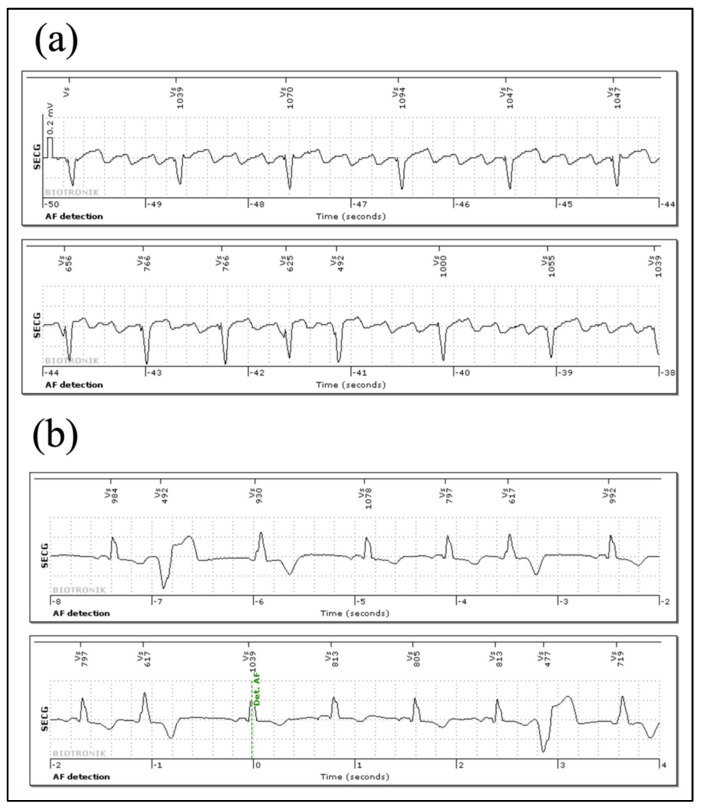
Subcutaneous ECG examples: (**a**) Correct classification as atrial fibrillation in a subcutaneous ECG showing atrial flutter; clear visualization of the P-wave improves diagnosis, and may guide the treatment. (**b**) Frequent premature ventricular contractions (PVCs); irregular R–R intervals due to ectopic beats can be misinterpreted as the start of an AF episode.

In total, a median of 99 days (IQR: 92–107) were analyzed. Of the 30 patients included in the study, 5 patients used Home Monitoring^®^. Through Home Monitoring^®^ transmission and in-house follow-up, a total of 408 true episodes were stored in the episode counter in 14/30 patients (47%). In 16 (53%) patients, no true events were recorded. The median number of subcutaneous ECGs (sECGs) per patient was 6 (IQR: 1.5–52.5; patients with no events excluded). The episodes consisted mainly of bradycardia (42%) and AF (29%). In two patients, more episodes (275 and 954) were detected during the follow-up period than the ICM could store. Since reaching the maximum storage volume of the ICMs resulted in overwriting of stored sECGs, not every episode was accessible and, therefore, could be analyzed independently. On the other hand, in total, 9817 misclassifications were detected in 13/30 (43%) patients. The median number of misclassifications was low (8.5; IQR: 3.5–20.5; patients with no events excluded). The overall high number of misclassifications was mainly recorded in two patients with 1571 and 8154 episodes, respectively. The main reasons for misclassification were P-wave oversensing, undersensing of premature ventricular contractions (PVCs), and misclassification of repeated premature atrial contractions (PACs) or PVCs as AF. Tracings of correctly annotated atrial flutter and PVCs misclassified as AF are shown in Figure 3.

### 3.5. Follow-Up and Therapeutic Consequences

In 10/30 (33%) patients, therapeutic interventions were performed based on the data of the ICM, including pacemaker implantation in 5 (50%) patients, ICD implantation in 1 (10%) patient, pulmonary vein isolation in 2 (20%) patients, and electrophysiological study in 2 (20%) patients. 

### 3.6. Questionnaire and Patient Comfort

**Figure 4:**
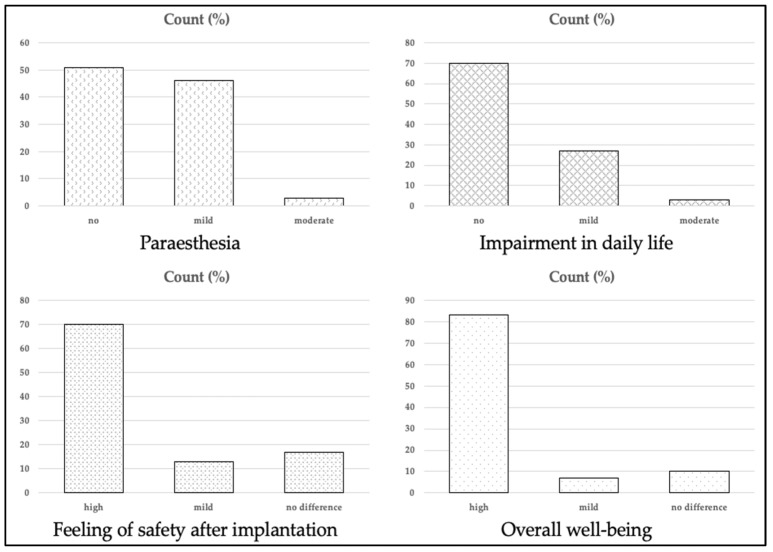
Evaluation of questionnaire answers provided by the study population.

At the first follow-up visit, all patients completed the questionnaire. Three patients (10%) reported moderate-to-severe pain and 13 (43%) patients reported mild pain at the implantation site within 24 h post-intervention. All other patients (47%) did report no relevant pain post-implantation. One (3%) female patient reported persistent discomfort of the ICM due to its position relatively close to the breast. Sustained paresthesia was moderate in 1 (3%) patient and mild in 14 (46%) patients, and was not reported in 51% of patients. All patients who were given a Home Monitoring^®^ system reported that installation was very easy. None of the patients needed help with the installation of the system. After the implantation, 21 (70%) felt safer, 4 (13%) felt partially safer, and only 5 (17%) did not report any difference. Additionally, fear of the next event was reduced in 10 (33%) and partially reduced in 8 (27%). The remaining 12 (40%) patients reported still being afraid of another event. Impairment in daily life was reported to be moderate by 1 patient (3%) and mild in 8 (27%) patients, whereas 21 (70%) patients reported no impairment. The cosmetic result was very satisfying in 21 (70%) patients, and satisfying in 5 (17%) of the patients; only 4 (13%) patients were less satisfied with the cosmetic result. This resulted in a high wellbeing in 25 (83%) patients and mildly improved wellbeing in 2 (7%) patients. Three (10%) patients did not report any improvement in their wellbeing. A selected number of questions of the questionnaire are visualized in Figure 4. 

## 4. Discussion

The new BIOMONITOR III ICM was developed to facilitate a minimally invasive procedure and improve the sensing quality and arrhythmia detection by a long sensing vector. Our prospective, single-center study demonstrated a high sensing quality and detection performance as well as a stable anatomical position of the ICM in a real-world setting. Using a newly designed insertion tool, we could successfully insert the ICM in a quick and simple procedure of less than 10 min in all 36 patients. 

The current generation of ICMs are smaller and easier to implant [10]. The perceived benefits of continuous long-term ECG monitoring have led to an increase in the number of ICMs being implanted. In line with the current guidelines, which have strengthened the indications for ICM implantation, the use of ICMs is expected to further increase over the next years. The improved diagnostic power, remote monitoring, and a longevity of up to 4 years further support this trend [11,13,14,15]. Possible indications for implantation are suspected arrhythmias, embolic stroke of unclear origin, recurrent syncope, or post-myocardial infarction in patients at risk of ventricular arrhythmias [9,14,16]. The ability of an ICM to detect an arrhythmia correctly is critically dependent on its ability to reliably sense the R-wave. Since the ICMs show a trend towards downsizing, R-waves’ amplitudes may be decreased, resulting in a higher amount of undersensing. Improved sensing quality can help to avoid signal dropout and undersensing that might lead to missed true arrhythmia and a high time burden of caring physicians to adjudicate these detections. The antenna and long sensing vector of the ICM are intended to provide large R-wave amplitudes and reliable sensing. In our study, R-wave-sensing was on average 0.7 mV after implantation, with an increase after 3 months (0.81 mV). Even the lowest measured values of less than 0.3 mV in 2/30 (6%) patients were sufficient for reliable sensing in our cases. Previously, an R-wave of at least 0.3 mV was recommended to be necessary [17]. Our sensing data are in line with the first-in-human investigation of the BIOMONITOR III ICM and its precursor model, the BIOMONITOR II, which also demonstrated R-wave sensing between 0.7 mV and 0.85 mV, respectively [3,11,18]. In other currently available ICM models without a long-vector design, the mean R-wave amplitudes did not exceed 0.6 mV [19,20,21]. This may possibly decrease the arrhythmia detection accuracy [20,22,23]. In obese patients, for example, the R-wave amplitude may decline to a level that is considered an absolute minimum for an adequate detection [17]. The long sensing vector of the ICM may be of additional value in these cases.

Patients with higher sensed R-waves were less likely to have a high noise burden. In our study, the noise burden was less than 2%, which is close to the range that was reported previously by Mariani et al. [11], and is very low in our opinion. Noise, such as electromagnetic interference or muscle potential, precludes a rhythm classification to avoid false positives and data overflow. A high noise burden thus decreases the diagnostic value of the ICM. Thus far, the BIOMONITOR ICMs are the only ones to count the noise burden rather than merely suspending the cardiac rhythm classification in the presence of noise without quantifying it.

Moreover, an anatomically stable body position is crucial for stable sensing parameters. Various studies have reported that the R-wave sensing amplitude might vary depending on the body position, which can possibly lead to undersensing. Even in- and expiration may alter the R-wave sensing [24,25]. In the BIOMONITOR II, we previously analyzed the anatomical stability by measuring the ICM body position during implantation and follow-up examination [12]. In this study, we were also able to demonstrate a stable anatomical position with no relevant repositioning, even with the now miniaturized profile of the ICM. It is not necessary to fixate the ICM, as was common in previously implanted ICMs such as the Medtronic Reveal XT or the St. Jude Medical Confirm. The recommended positions of the ICM are (a) along the heart axis or (b) parasternal, because of the most proximal position to the heart to guarantee sufficient sensing. In one young female patient with a BMI of less than 20 kg/m^2^_,_ the ICM was implanted in an axillary position to improve the aesthetic result, resulting in an equivalent sensing performance of 1.47 mV after 3 months. For selected patients, an alternative position should be preferred in order to increase acceptance and reduce unnecessary fear of this useful and low-risk technology. Bisignani et al. already demonstrated an axillary insertion to be a valid alternative to the standard one for long-dipole ICM technology, providing not only patient acceptability but also high-quality sensing performances [26].

In this study, the ICM demonstrated high diagnostic accuracy, based on a high sensing performance and a low noise burden. In 43% of our patients, misclassification occurred. However, the total number was mainly determined by two patients with a very high number of false positives. These were mainly caused by PVC undersensing and misjudgment of repeated PACs or PVCs as AF. This false positive AF detection along with the data overload in two patients requires careful assessment of the episodes, as this could significantly impact clinical treatment decisions. Remote monitoring may further help to prevent loss of relevant episodes in case of multiple detections, and may prevent delays in patient care—especially in ICM patients, who are otherwise well suited for exclusive remote follow-up. Further improvements in detection algorithms, understanding of possible limitations, and optimal ICM programming are required in order to optimize the potential clinical utility of ICM-based ECG monitoring. The BIOMONITOR IIIm may help to overcome this issue with the new RhythmCheck algorithm by eliminating false AF episodes in patients with frequent ectopic beats. In addition, incorporating P-wave information in the ILR algorithm may significantly reduce the number of inappropriately detected AF episodes. An individual programming or usage of the predefined, indication-related programming may also add to the diagnostic accuracy. However, since we have not made any programming changes, no statement can be made regarding any improvement through these changes.

Downsizing of the ICM should simplify the implantation procedure and increase patient satisfaction. Most of our patients were satisfied after implantation, reporting a high wellbeing with the ICM. The overall high wearing comfort is consistent with the larger predecessor, the BIOMONITOR II ICM [12]. This satisfaction is due to a very good cosmetic result, an improvement in the safety of the continuous monitoring, and a low pain burden from the implanted ICM. We attribute the continuing fear after implantation to the fear of another event, and do not consider this to be device-related. Only one female patient reported persistent discomfort due to the ICM’s position relatively close to the breast. In one female 90-year-old patient, the device protruded 2 weeks post-implantation—probably because of distinct cachexia. As most patients were satisfied with the cosmetic result, the size of the ICM does not seem to be of paramount importance. Further downsizing of the ICM may therefore only have a minor impact on patient comfort, but may impair sensing quality and longevity. We therefore consider an alternative axillary insertion as an option in the event of challenging anatomy, with the aim of avoiding complications and improving patient acceptance [26].

### Limitations

One limitation of this study is the small patient cohort and the follow-up of only three months. Moreover, due to limitations in storage capacity, not every sECG was accessible. Comparison to other ICMs is not possible. This study was conducted in a single center, and this may affect the validity of the data regarding the insertion procedure.

## 5. Conclusions

The results of our study show that implantation of the novel BIOMONITOR III ICM is fast and uncomplicated. The ICM demonstrates excellent sensing characteristics, even improving over time, and patient satisfaction is high.

## Figures and Tables

**Table 1:** Baseline characteristics.

Age	67 ± 12
Sex (male)	12 (40%)
BMI (kg/m^2^)	26 ± 4
Coronary artery disease	6 (20%)
Hypertension	17 (57%)
Diabetes mellitus	3 (10%)
Stroke	6 (20%)
Atrial fibrillation	5 (16%)
LVEF (%)	58 ± 5
CV drug use	
- Anticoagulation	6 (20%)
- Platelet aggregation inhibitors	11 (36%)
- ACE-inhibitors/AT1-antagonists	14 (46%)
- Beta blockers	12 (40%)
- Anti-arrhythmics	0 (0%)

Data are presented as the mean ± standard deviation. Abbreviations—BMI: body mass index; LVEF: left ventricular ejection fraction; CV: cardiovascular.

**Table 2:** R-wave sensing parameters.

	n=	Mean ± SD	Range
R-wave sensing			
Post-op	30	0.73 ± 0.32 mV	0.27–1.47 mV
Day 1	30	0.78 ± 0.38 mV	0.25–1.52 mV
Follow-up	30	0.81 ± 0.39 mV	0.20–2.00 mV

Data as mean ± standard deviation. Abbreviations: mV: millivolt.

## Data Availability

Not applicable.

## Abbreviations and Acronyms

Injectable cardiac monitor	ICM
Electrocardiographic	ECG
Atrial fibrillation	AF
Fast insertion tool	FIT
Subcutaneous ECG	sECG
Premature ventricular contractions	PVC
Premature atrial contractions	PAC
